# Establishment and evaluation of a UPLC-MS/MS method for simultaneous determination of bongkrekic acid and dehydroacetic acid in rice noodles

**DOI:** 10.3389/fchem.2024.1386635

**Published:** 2025-01-28

**Authors:** Lingguo Zhao, Ronggui Lv, Jiang Song, Xi Chen, Lei Lei, Weixian Zeng, Miaowen She, Dabing Li, Xiaxia Yu, Zhanguo Liu, Xiao Wang, Yong Liu

**Affiliations:** ^1^ Department of Physical and Chemical Laboratory, Center for Disease Prevention and Control of Baoan District, Guangdong, Shenzhen, China; ^2^ Department of Critical care medicine, Shenzhen Hospital, Southern Medical University, Guangdong, Shenzhen, China; ^3^ Department of Critical Care Medicine, Zhujiang Hospital, Southern Medical University, Guangzhou, China; ^4^ Department of Ultrasonography, Shenzhen Prevention and Treatment Center for Occupational Diseases, Shenzhen, China; ^5^ Department of Critical Care Medicine, Taihe Hospital, Hubei University of Medicine, Shiyan, Hubei, China; ^6^ Accident and Emergency Department (AED), Kiangwu Hospital, MacaoSAR, China; ^7^ School of Biomedical Engineering, Health Science Center, Shenzhen University, Shenzhen, China; ^8^ Research Centre of Basic Integrative Medicine, School of Basic Medical Sciences, Guangzhou University of Chinese Medicine, Guangzhou, Guangdong, China

**Keywords:** bongkrekic acid, dehydroacetic acid, UPLC-MS/MS, burkholderia gladiolus, rice noodles

## Abstract

A UPLC-MS/MS method with one-step extraction and simultaneous determination of bongkrekic acid (BA) and dehydroacetic acid (DHA) in rice noodles has been established in this study for the first time. The extraction solvent consisted of a mixture of methanol, ammonia, and water. Electrospray ionization negative ion mode (ESI-) was used for multiple reaction detection and external standard method was used for quantitative determination. The method demonstrated a good linearity range of 0–50 ng/mL with a correlation coefficient (r) of 0.9998 for BA, and showed a linearity range of 0∼500 ng/mL with r of 0.9993 for DHA. The limits of detection (LOD) were determined to be 0.1 μg/kg for BA and 0.6 μg/kg for DHA. Additionally, the method exhibited good recovery of 90.1%∼105.4% for BA and 80.4%∼102% for DHA. Meanwhile, the method also exhibited good precision with RSD of 0.4%∼7.5% for BA, and with RSD of 4.5%∼11.4% for DHA. Furthermore, the established method was sucessfully applied to the analysis of rice noodles from two production enterprises. BA was detected in one rice noodle sample and DHA was detected in 7 rice noodle samples in the enterprises A which was the manufacturer of food consumed by the poisoned patient. To investigate the cause of death of the poisoned patient, the toxigenic bacterium *Burkholderia gladiolus* was isolated and cultured to produce BA. The results showed that the established method could also be used to qualitatively screen BA and its isomer isobongkrekic acid (IBA) in GVC enrichment broth, potato glucose agar and rice sample, which facilitates a rapid and accurate judgement of whether the isolated bacterium strain produces BA. With high sensitivity and accuracy, and simple sample pretreatment, the proposed method shows potential for safety assessment of the entire production process of rice-derived products, addressing a significant public health concern.

## 1 Introduction

Bongkrekic acid (BA) is a mitochondrial toxin that inhibits adenine nucleotide translocase. It is a tricarboxylic fatty acid produced by the bacterium *Burkholderia gladiolus* pathovar *cocovenenans* (Li et al., 2019). Before 2015, food poisoning caused by this bacteria was only reported in China and Indonesia in Asia (Anwar et al., 2017). Indonesia has reported that cumulatively, more than 9,000 people have been affected and more than 1,000 have died since the end of the 19th century. The main cause was spoiled and fermented coconut products (Anwar et al., 2017; Garcia et al., 1999). Since the mid-20th century, China has reported a cumulative number of more than 2,000 cases, with nearly 1,000 deaths primarily from spoiled and fermented corn products (Anwar et al., 2017). In 2015, an outbreak of BA poisoning due to consumption of the traditional African beverage that is made mainly with corn was reported in Mozambique, Africa, resulting in 75 deaths and 177 hospitalizations (Gudo et al., 2018). In 2018, a family in Heyuan city, Guangdong province, China experienced food poisoning. Upon investigation, it was determined that the poisoning was caused by the consumption of rice noodles contaminated with BA, despite the food not being noticeably fermented or spoiled (Li et al., 2019). Recent studies have highlighted the critical nature of BA poisoning, with cases involving severe liver function damage and multiple organ failure reported after the consumption of spoiled food (Shi et al., 2019). The toxicokinetic behavior and removal of BA during blood purification therapies have also been investigated in our previous work (Lv et al., 2023). Furthermore, the identification of BA in traditional African beverages linked to fatal outbreaks underscores the global impact of this toxin (Falconer et al., 2017). High-performance liquid chromatography (HPLC) is the primary method for determining BA in food samples (Voragen et al., 1982), the selectivity and sensitivity are low, making it challenging to characterise samples with low concentrations. A pre-concentration procedure using Fe_3_O_4_/HNTs (Fe_3_O_4_ attached on Halloysite nanotubes) was developed to adsorb BA and its cis-trans isomers isobongkrekic acid (IBA) in rice noodles and then the extracts were analysed by HPLC-Orbitrap HRMS (Liang et al., 2021). PRiME-UHPLC-MS/MS method was also reported to quantify aflatoxins and bongkrekic acid in rice and noodle products using SPE purification and QuECHERS clean-up procedure for the pretreatment (Hu et al., 2022). Cysteamine-stabilized gold nanoparticles (CS-AuNPs) were used as colorimetric probes to visually detect BA in a dual-mode platform based on UV-Vis absorption (Zhang et al., 2022). A dispersive liquid-liquid microextraction method was established for determination of BA in plasma by LC-MS/MS (Xu et al., 2021). However, these methods have several limitations. The special synthetized materials such as Fe3O4/HNTs (Liang et al., 2021) and CS-AuNPs (Zhang et al., 2022) were needed in these methods, or the complex and time-consuming pretreatment procedure requiring several different organic solvents were adopted in these methods such as SPE (Hu et al., 2022), QuECHERS (Hu et al., 2022) and liquid-liquid microextraction (Xu et al., 2021). Meanwhile, the dehydroacetic acid could not be detected simultaneously.

Dehydroacetic acid (DHA) and its sodium salt are powerful preservatives that effectively inhibit fungi, yeasts, and bacteria. This makes them an ideal choice for preventing the alteration and degradation of food by microorganisms (Spencer et al., 1950). To date, DHA and sodium dehydroacetate are permitted in the United States, China, Korea, and Japan under specific legislation on food additives (Ohtsuki et al., 2013). In China, DHA is approved for use with a maximum amount of no more than 100 mg/kg (National Health Commission of the People’s Republic of China, 2024d). The addition of DHA to rice noodles is intended to inhibit mold growth, which could inadvertently conceal the presence of BA. This might lead to a false sense of security regarding the safety of the rice noodles, as the absence of visible mold does not guarantee that the product is free from BA contamination. Moreover, it poses a potential risk by preventing rice noodles from moulding and spoiling, leading operators and consumers to mistakenly believe that long-lasting rice noodles possible with BA is safe to eat, even it is not (Li et al., 2019). BA and DHA are important and potentially interrelated control points for rice-derived products, and it is necessary to establish a method to allow for simultaneous testing. Therefore, it is vital to analyze and monitor the BA and DHA in rice-derived products simultaneously, even if the sample does not ferment or noticeably spoil. Currently, the official methods for DHA detection include liquid chromatography (LC) and gas chromatography (GC) as outlined in China’s national food safety standards GB 5009.121-2016 (National Health Commission of the People’s Republic of China, 2024b). Specifically, liquid-liquid extraction (LLE) was adopted for GC analysis in the first method, and SPE coupled with LC analysis was used in the second method (National Health Commission of the People’s Republic of China, 2024b). Moreover, there are some reports on DHA detection using GC-MS (Kakemoto, 1992) and quantitative 1H NMR (Ohtsuki et al., 2013). So far, LC-MS/MS method for the determination of DHA has not been reported in the literature. In this work, a straightforward approach for the simultaneous determination of bongkrekic acid and dehydroacetic acid in rice noodles by LC-MS/MS was reported for the first time.

## 2 Materials and methods

### 2.1 Instruments and reagents

BA standard solution in methanol (1.0 mg/mL) was purchased from Shanghai Anpu Experimental Technology Co., Ltd. (Shanghai, China). Dehydroacetic acid standard solution in methanol (1.0 mg/mL) was purchased from Beijing Tanmo Quality Inspection Technology Co., Ltd. (Beijing, China). Methanol and acetonitrile of HPLC grade were obtained from Thermo Fisher Scientific (Fisher, United States). Acetic acid of analytical grade was purchased from Shanghai Aladdin Biochemical Technology Co., Ltd. Nylon membrane filters with pore size of 0.2 μm were supplied by Tianjin Fuji Science and Technology Co., Ltd. Rice noodles were obtained from local supermarkets as blank sample. The rice, Semi-finished Rice Noodles and Finished Rice Noodles were obtained from Enterprise A and B. GVC enrichment broth and Potato Dextrose Agar (PDA) were procured from Guangdong Huankai Microbial Technology Co., Ltd.

The AB SCIEX QTRAP 5500 mass spectrometer (AB SCIEX, United States) was utilized for the determination of BA and DHA. For the centrifugation of sample solutions, The Sigma 3-18 KS centrifuge (Sigma, Germany) was utilized in the study. A high-performance dispersing instrument IKA T-25 (IKA Works, Germany) was utilized to ensure a homogeneous sample matrix before analysis. Ultrasound cleaning instrument KQ-700E (KunShan Ultrasonic Instrumnet, China) was used for the extraction of samples.

### 2.2 Chromatographic and mass spectrometry conditions

Analysis was performed using an ACQUITY UPLC system (Waters Corp., Milford, MA, United States) coupled with SCIEX QTRAP 5500 mass spectrometer. The chromatographic separation was achieved using a Waters ACQUITY UPLC CSH C18 Column (2.1 mm × 100 mm i. d., 1.7 µm particle size, 130 Å pore size) kept at 40°C in the oven. The flow rate was 300 μL/min and the injection volume was 5 µL. Mobile phase A (0.2% acetic acid in water) and mobile phase B (acetonitrile with 0.2% acetic acid) were used for the gradient elution. The program was as follows: 0∼1 min, 20%B; 1∼6 min, 20%B-100%B; 6∼10 min, 100%B; 10∼11 min, 100%B-20%B; 11∼13 min, 20%B。

The MS condition was listed as follows: ionspray voltage was −4,500 V, source temperature was 550 C, nebulization and heating gas was 55 psi, curtain gas was 35 psi. Electrospray ionization (ESI) with negative mode and Multiple Reaction Monitoring mode (MRM) were used in the method. MRM transitions and optimized parameters were listed in Table 1.

**TABLE 1:** MRM parameters of BA and DHA.

Compound	Q1 Mass (Da)	Q3 Mass (Da)	Ce (volts)
BA	485.4	441.4^a^	−15
		397.3	−25
		379.1	−27
		365.3	−29
		321.4	−27
DHA	167.0	123.1	−12
		83.0^a^	−22
		81.0	−26

^a^ Numbers in bold with an asterisk indicate the most abundant and stable product ions selected for the quantification of BA, and DHA.

### 2.3 Sample preparation

#### 2.3.1 Rice-derived production samples

The ammonia methanol solution was prepared by mixing 1 mL of ammonia, 80 mL of methanol and 19 mL of water. Rice-derived production sample (about 5.00 g) was accurately weighed and mixed with 20 mL of ammonia methanol solution. The mixture was then homogenized and vortexed for 10 min. Subsequently, the samples were extracted using ultrasonication for an additional 20 min at 40 C with the frequency of 40 kHz. Then, the samples were centrifuged at a temperature of 4 C and 10,000 g for 5 min. The supernatant was filtered through 0.20 μm filter membrane for analysis. The blank matrix solution was prepared in the same way and then used for the standard curve preparation.

#### 2.3.2 GVC enrichment broth

The GVC enrichment broth sample was prepared by adding 800 μL of ammonia methanol solution into 200 μL of GVC enrichment broth. Then the mixture was vortexed for 1 min and filtered through a 0.2 μm filter membrane for sample analysis. GVC enrichment broth is a commercial liquid medium for selective enrichment culture for *B. gladiolus.* It is composed of potato powder, glucose, crystal violet, and chloramphenicol.

#### 2.3.3 Potato dextrose agar

The potato dextrose agar sample was prepared by adding 200 μL of the melt potato dextrose agar into 10 mL of ammonia methanol solution, and then the mixture was shaken evenly. The nylon membrane filter with pore size of 0.2 μm was adopted to filter 1 mL of mixture, and subsequently the filtrate was injected into the UPLC-MS system for analysis. Potato dextrose agar is a commercial solid medium for the production of BA and IBA by *B. gladiolus* in this study.

### 2.4 Standard curve preparation

BA standard solution (1.0 mg/mL) and DHA standard solution (1.0 mg/mL) were diluted to prepare 100 ng/mL BA stock solution and 1,000 ng/mL DHA stock solution with blank matrix solution respectively. Working solutions of lower concentrations were then prepared by diluting these stock solutions using blank matrix solution, with concentrations of 0, 3, 5, 10, 20, 30, 40, and 50 ng/mL for BA, and 0, 30, 50, 100, 200, 300, 400, and 500 ng/mL for DHA. Quantification of BA and DHA was performed using the external standard curve method. A weighted least-squares linear regression analysis was used to calculate the regression equation for peak area ratios in relation to their respective concentrations.

### 2.5 Limit of quantification and limit of detection

BA standard solution (1.0 μg/mL) and DHA standard solution (1.0 μg/mL) were added into 5.00 g of blank sample, the spiked volume of both standard solutions was 100 μL. Then the spiked sample was treated and analyzed using the method described in Section 2.3.1.

The limit of detection (LOD) and the limit of quantification (LOQ) are determined by the signal to noise approach. A signal to noise ratio of 3:1 was used to estimate the detection limit and 10:1 to estimate the quantification limit.

### 2.6 Recovery and reproducibility

BA and DHA standard solution were added into nine blank samples to create three groups of spiked samples (low, medium and high concentration group). Each group was prepared in triplicate. The low, medium and high concentration group were spiked with 200, 500 and 1,000 μL of BA and DHA standard solution (1.0 μg/mL) respectively. The spiked samples were then treated and analyzed using the method described in Section 2.3.1.

## 3 Results and discussion

### 3.1 Qualitative and quantitative mass transitions

**FIGURE 1:**
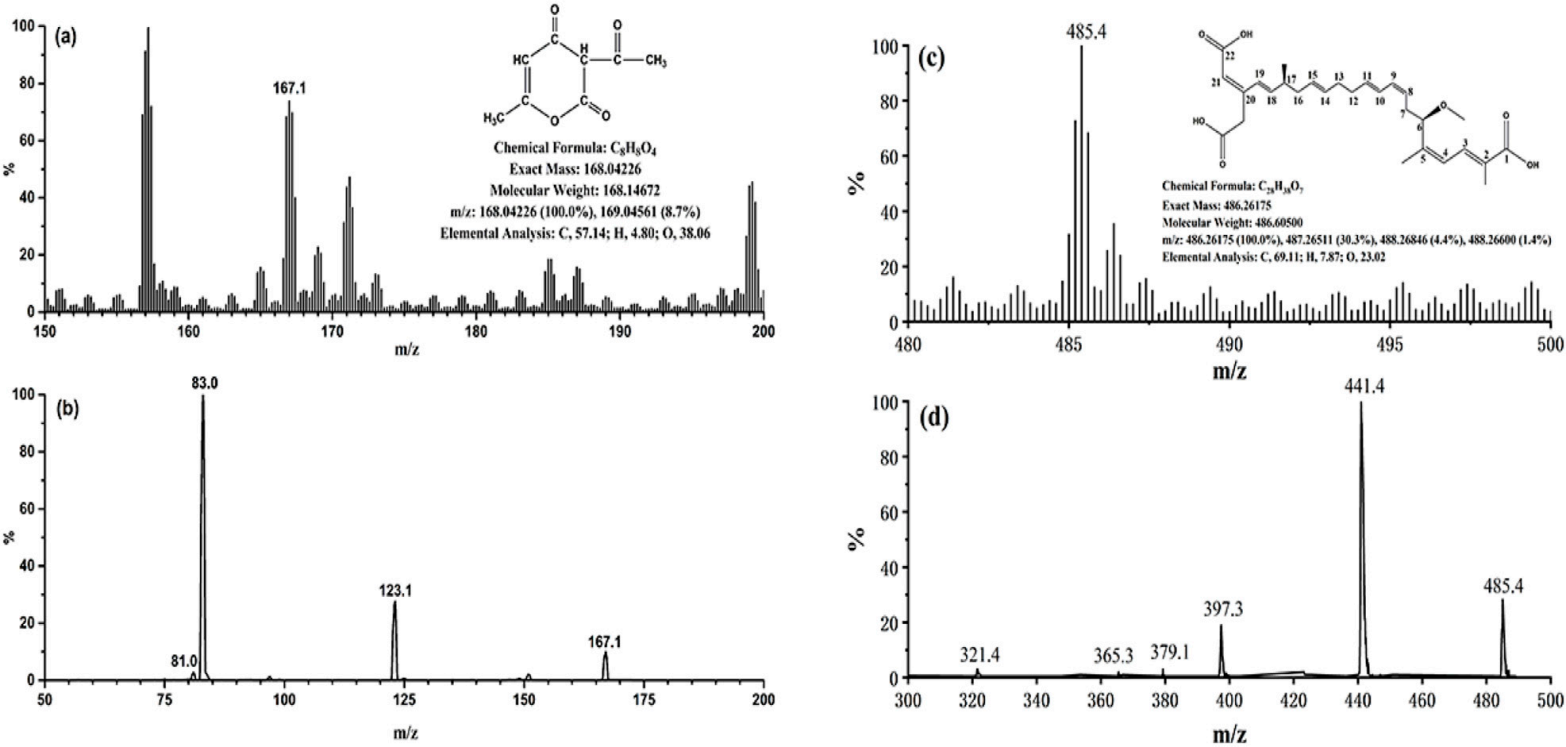
**(A)** Full scan mass spectrum of DHA; **(B)** MS2 scan of DHA; **(C)** Full scan mass spectrum of BA; **(D)** MS2 scan of BA.

The mass spectra of BA and DHA were presented in Figure 1. The precursor ion selected in the full scan spectra was the most abundant ion, corresponding to the ion [M-H]^-^ for BA and DHA. Collision energy and decluttering potential were automatically optimized. Table 1 lists the MRM transitions and optimized parameters. The mass transitions in bold were used for quantification.

### 3.2 Investigation of linear relationship

**FIGURE 2:**
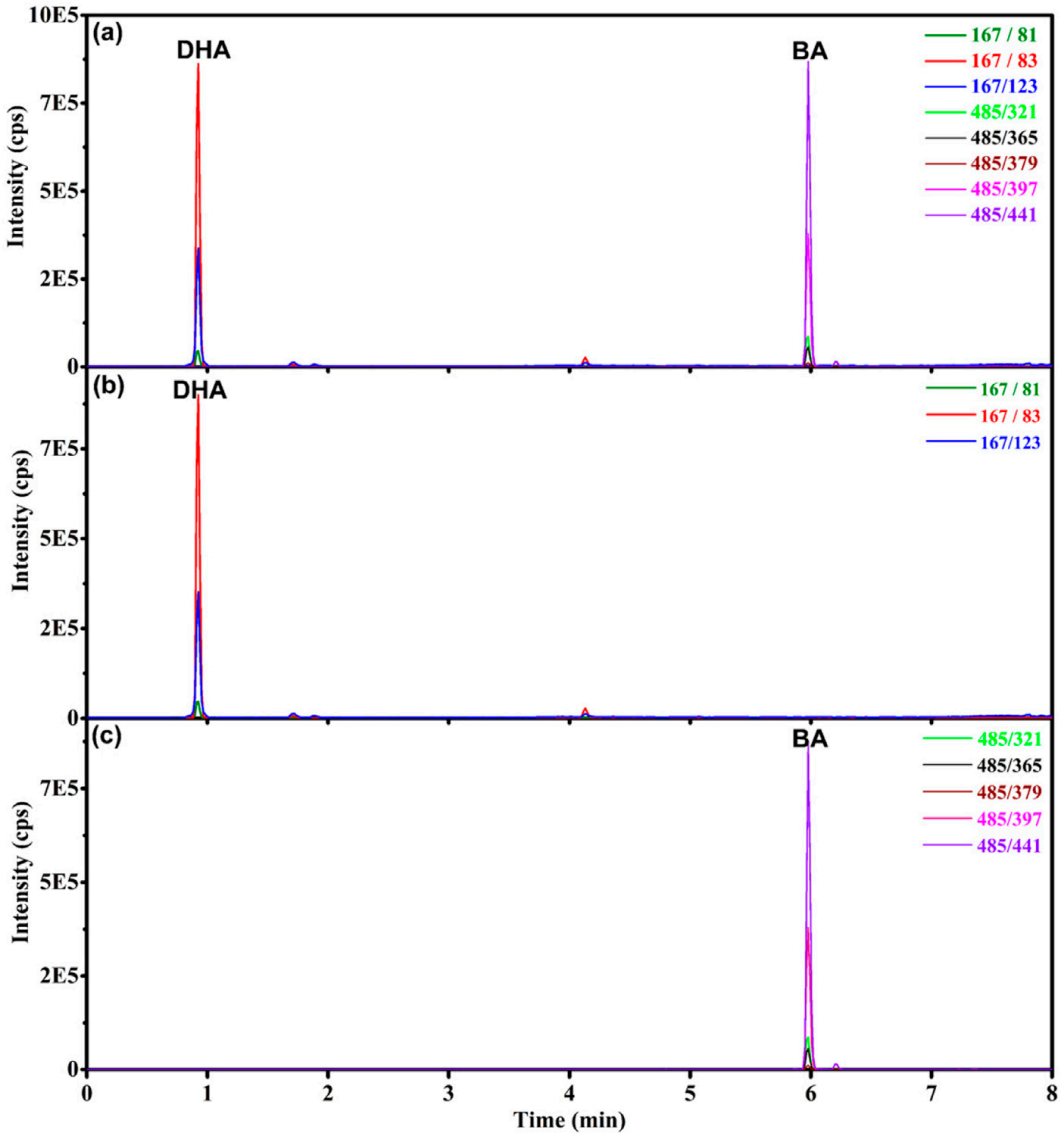
Chromatograms of BA and DHA standard solution. **(A)** Total ion chromatogram of DHA (500 ng/mL) and BA (50 ng/mL). **(B)** Extracted ion chromatogram of DHA (500 ng/mL). **(C)** Extracted ion chromatogram of BA (50 ng/mL).

The total ion chromatogram (TIC) and extracted ion chromatogram (XIC) of DHA and BA standard solution were shown in Figure 2. The mass transitions of BA and DHA are coaxial with symmetrical and sharp peaks, and good resolution. For BA, the linear regression analysis of the calibration plot data revealed a linear relationship between peak area and concentration over the range of 0∼50 ng/mL, with correlation coefficient (r) of 0.9998. The linear regression equation was *y* = 3.47 × 10^4^ *x*−3.55 × 10^3^, weighting: 1/*x*. For DHA, the linear regression analysis of the calibration curve data showed a linear relationship between peak area and concentration over the range of 0∼500 ng/mL, with correlation coefficient (r) of 0.9993. The linear regression equation was *y* = 3.26 × 10^3^ *x* + 8.71 × 10^3^, weighting: 1/*x*.

### 3.3 LOQ and LOD

As shown in Table 2, the limit of detection (LOD) and the limit of quantification (LOQ) for BA in this method were 0.1 μg/kg and 0.3 μg/kg, respectively. These values represent a substantial decrease in comparison to the LOD at 5 μg/kg and the LOQ at 15 μg/kg as specified in the HPLC method of China’s national food safety standards (GB 5009.189-2016) (National Health Commission of the People’s Republic of China, 2024a). Similarly, the LOD and LOQ for DHA in this method were determined to be 0.6eμg/kg and 2.0 μg/kg, respectively. These values were significantly lower than those specified in the HPLC method of China’s national food safety standards (GB 5009.121-2016) (National Health Commission of the People’s Republic of China, 2024b), which sets the LOD at 2 mg/kg and the LOQ at 5 mg/kg, indicating a greater sensitivity of this method.

**TABLE 2:** LOD and LOQ of the method.

Compound	Sample weight (g)	Spike content (μg/kg)	Signal to noise ratio	LOD (μg/kg)	LOQ (μg/kg)
BA	5.505	18.2	569.3	0.1	0.3
DHA		18.2	90.2	0.6	2.0

### 3.4 Recovery and precision of the method

The experimental results of recovery and precision assessment were shown in Table 3. The samples were randomly divided into three groups (n = 3/group). For both BA and DHA, the recoveries ranged from 80.5% to 105.4%. The relative standard deviation (RSD) in the low, medium, and high concentration groups demonstrated the precision of the method. For DHA, the RSD values were 11.4%, 6.9%, and 4.5%, respectively, indicating excellent precision at varying concentration levels. Similarly, for BA, the RSD values were within an acceptable range from 0.4% to 7.5%, showcasing the method’s reliability in quantifying both BA and DHA in the samples.

**TABLE 3:** Recovery and precision of the method.

Sample weight (g)	Spiked concentration (μg/kg)	DHA measured content (μg/kg)	DHA recovery (%)	DHA RSD (%)	BA measured content (μg/kg)	BA recovery (%)	BA RSD (%)
5.506	36.3	29.2	80.5	11.4	32.7	90.1	7.5
5.898	33.9	33.0	97.5		34.9	102.9	
5.639	35.5	28.5	80.4		36.5	102.8	
5.195	96.2	94.7	98.4	6.9	99.5	103.4	4.8
5.865	85.3	78.8	92.4		81.9	96.1	
5.700	87.7	75.1	85.7		92.4	105.4	
5.795	172.6	176.1	102.0	4.5	170.1	98.6	0.4
5.210	191.9	179.8	93.7		188.1	98.0	
5.509	181.5	173.3	95.5		179.0	98.6	

### 3.5 Application of the developed method

A 27-year-old man died of BA poisoning after eating rice noodle from a fast-food restaurant near his workplace (Lv et al., 2023). The rice noodle was produced by enterprise A and detailed investigation and sampling were conducted on enterprise A and another rice noodle production enterprise B to avoid similar BA poisoning events.

The assay results were shown in Table 4. DHA was detected in all the semi-finished products and finished products of enterprise A which was the manufacturer of food consumed by the poisoned patient. Conversely, DHA was not detected in another enterprise B. Interestingly, the toxin BA was detected in the semi-finished rice noodles of enterprise A, but BA was not detected in the semi-finished and finished products of enterprise B, indicating that DHA and BA may be potentially interrelated influencing factors in poisoning events (Li et al., 2019).

**TABLE 4:** The assay results of rice noodles.

Sample number	Sample name	Enterprise code	BA (μg/kg)	DHA (μg/kg)
21SZ-YQ-SC-01	Semi-finished Rice Noodles	A	15.2	329
21SZ-YQ-SC-02	Semi-finished Rice Noodles	A	<0.1	260
21SZ-YQ-SC-03	Semi-finished Rice Noodles	A	<0.1	305
21SZ-YQ-SD-01	Finished Rice Noodles	A	<0.1	461
21SZ-YQ-SD-02	Finished Rice Noodles	A	<0.1	564
21SZ-YQ-SE-01	Finished Rice Noodles	A	<0.1	553
21SZ-YQ-SE-02	Finished Rice Noodles	A	<0.1	905
21SZ-HY-SC-01	Semi-finished Rice Noodles	B	<0.1	<0.6
21SZ-HY-SC-02	Semi-finished Rice Noodles	B	<0.1	<0.6
21SZ-HY-SC-03	Semi-finished Rice Noodles	B	<0.1	<0.6
21SZ-HY-SD-01	Finished Rice Noodles	B	<0.1	<0.6
21SZ-HY-SD-02	Finished Rice Noodles	B	<0.1	<0.6
21SZ-HY-SE-01	Finished Rice Noodles	B	<0.1	<0.6
21SZ-HY-SE-02	Finished Rice Noodles	B	<0.1	<0.6

**FIGURE 3:**
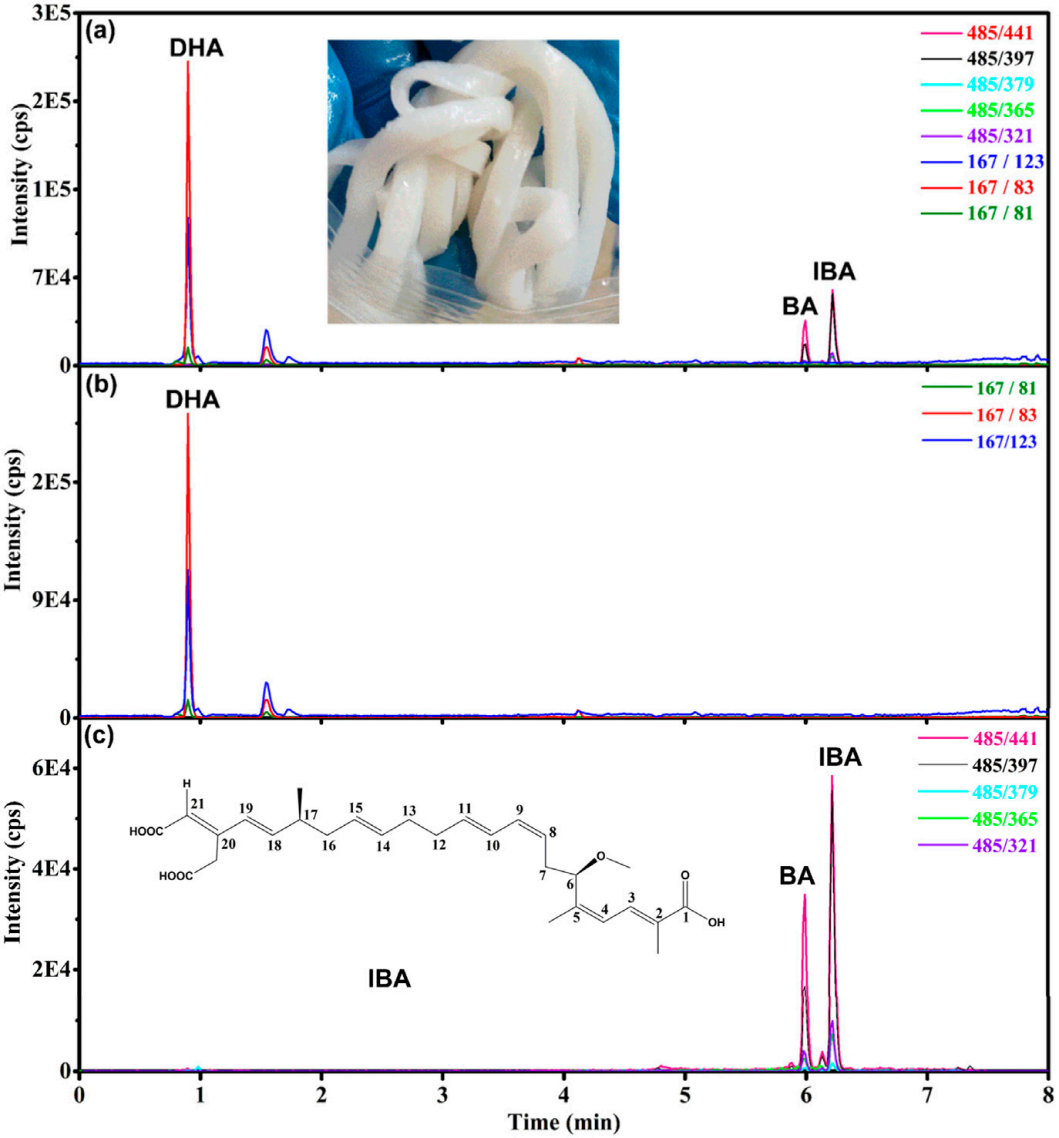
Chromatograms of dehydroacetic acid (DHA), bongkrekic acid (BA) and isobongkrekic acid (IBA) in rice noodle sample (21SZ-YQ-SC-01) with BA at a concentration of 15.2 μg/kg and DHA at 329 μg/kg. **(A)** Total ion chromatogram of sample. **(B)** Extracted ion chromatogram of DHA. **(C)** Extracted ion chromatogram of BA and IBA.

**FIGURE 4:**
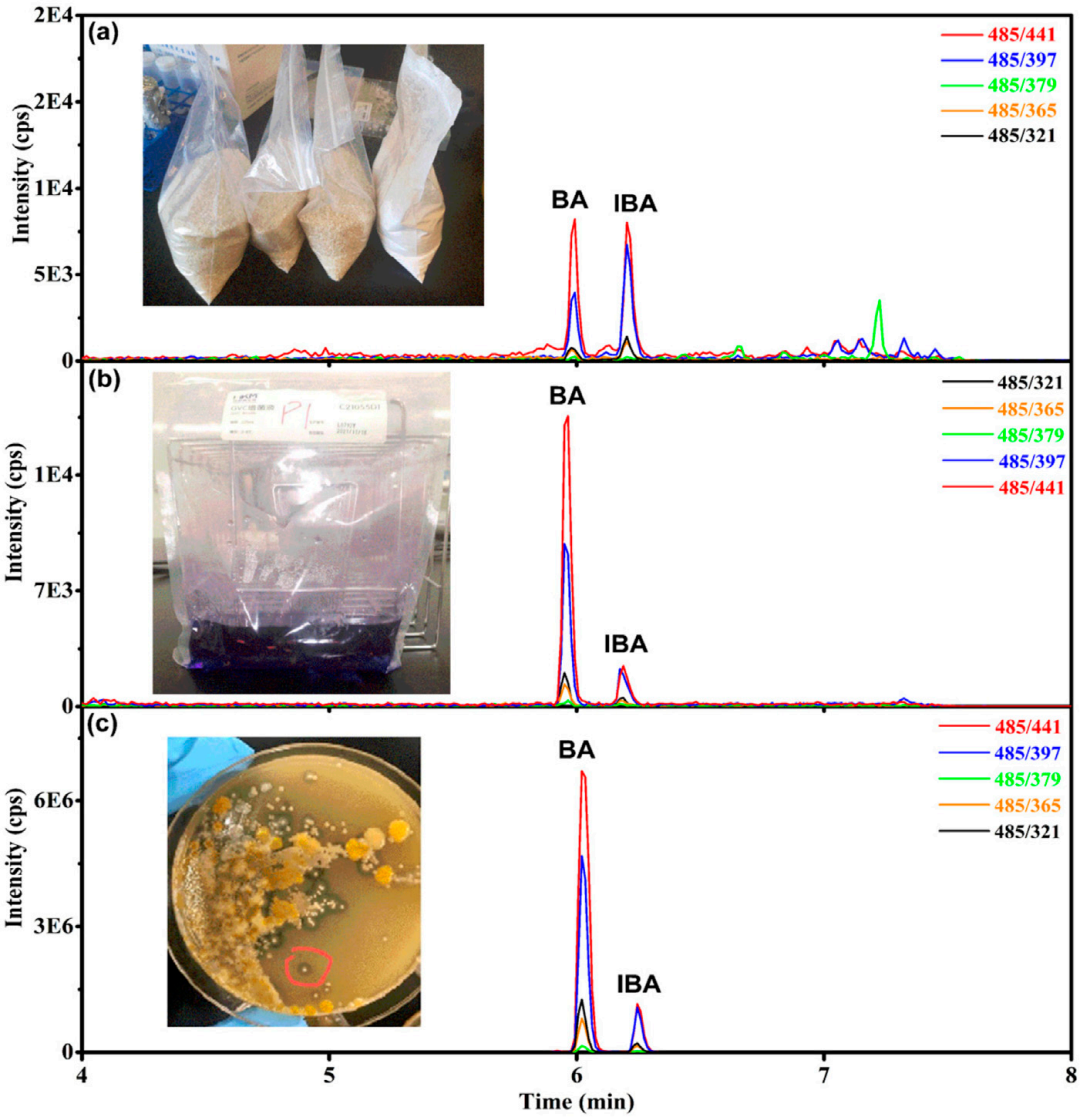
Chromatograms of bongkrekic acid (BA) and isobongkrekic acid (IBA) in different samples. **(A)** Extracted ion chromatogram of BA and IBA in rice. **(B)** Extracted ion chromatogram of BA and IBA in GVC enrichment broth. **(C)** Extracted ion chromatogram of BA and IBA in potato glucose agar.

The chromatogram of rice noodles sample (21SZ-YQ-SC-01) was shown in Figure 4. Different from the standard solution in Figure 2, isobongkrekic acid (IBA) appeared in the real sample in Figure 3. The extraction ion chromatogram (XIC) of BA displays two associated peaks, which correspond to BA and its isomer, isobongkrekic acid. These compounds are cis-trans isomers, differing in the configuration of their dicarboxylic ends. Specifically, BA features a trans configuration (Figure 1C), while IBA has a cis configuration at the dicarboxylic end (Figure 3C) (Lauquin et al., 1976). BA was unstable under light conditions and slowly converted to IBA (Chatterjee et al., 1988), so IBA and BA were associated in the real samples. They have the same fragment ions and MRM transitions as shown in Figure 3. The retention time could be used to distinguish BA from IBA. The compound with a shorter retention time is identified as BA, whereas those with longer retention times are IBA (Liang et al., 2021).

Furthermore, the study revealed that the ion ratios are different for BA and IBA. As illustrated in Figure 3, the 441 was the base peak (the highest peak), the 397 is the second highest peak. The ion ratio of 397/441 was 43% for BA, but it was 73% for IBA. This phenomenon can also be confirmed in Figure 4. The XIC of 441 was red and the XIC of 397 was blue, the ion ratio of 397/441 for IBA is significantly higher than that for BA. This finding facilitates the discrimination between BA and IBA isomers.

### 3.6 Methods for screening application

In this investigation, BA and IBA was detected in the semi-finished rice noodle (21SZ-YQ-SC-01) from enterprise A (Figure 3), indicating that the samples may be contaminated by toxigenic bacterium *B. gladiolus*. To investigate the cause of death of the patient, the toxigenic bacterium *B. gladiolus* was isolated from the rice noodle (21SZ-YQ-SC-01) and cultured in GVC enrichment broth as in Figure 4B. Then *B. gladiolus* was inoculated into potato glucose agar culture to produce BA as in Figure 4C according to the China’s national food safety standards GB 4789.29-2020 (National Health Commission of the People’s Republic of China, 2024c). Finally, potato glucose agar was melt and the mice were perfused with it. Whether BA was generated was judged by whether the mice were dead (National Health Commission of the People’s Republic of China, 2024c). This method in GB 4789.29-2020 was complex, ambiguous and time-consuming. In this study, the established quantitative method for BA and DHA in rice noodles could also be used to qualitatively screen BA and IBA in GVC enrichment broth, potato glucose agar and rice as in Figure 4. This facilitates a rapid and accurate judgement of whether the isolated strain produces BA. The accurate content of BA in GVC enrichment broth and potato glucose agar were not improtant for the judgement of toxic strains, thus the matrix effect and quantification method were not verified for GVC enrichment broth and potato glucose agar.

It was worth mentioning that *B. gladiolus* was reported to produces about 5% IBA at the same time as BA (Liang et al., 2021). In this study, it was found that the ratio of IBA to BA was low in the GVC enrichment broth and potato glucose agar as in Figures 4B,C, the ratio of IBA to BA was 12.6% and 15.5%, respectively. However, BA was reported to convert to IBA slowly under sunlight (Chatterjee et al., 1988), so it was found that the ratio of IBA to BA could be high as 96.4% in rice (Figure 4A) and 169% in rice noodles (Figure 3) in this study. It has been shown that the ratio of IBA to BA was related to the storage time of the contaminated material and production.

The amount of IBA in the purified BA standard solution was extremely low (Figure 2), so the presence of IBA in the sample can effectively rule out false positives caused by standard contamination. At the same time, the ratio of IBA to BA in the sample is proportional to the duration of contamination (poisoning), which can be used as indirect evidence for tracing the source of the toxin.

## 4 Conclusion

Although poisoning from BA has not been reported outside of Asia and Africa until recently, this does not exclude the presence of BA-associated illness in other parts of the world (Anwar et al., 2017). DHA prevent rice noodles and other food from moulding and spoiling, leading operators and consumers to mistakenly believe that long-lasting food possible with BA is safe to eat. A UPLC-MS/MS method with one-step extraction and simultaneous determination of BA and DHA in rice noodles has been established in this study for the first time with good accuracy and sensitivity. Meanwhile, the ion ratio of 397/441 was firstly reported to be significantly different for BA and IBA in this study and this finding facilitates the discrimination between BA and IBA isomers besides retention time. In addition, the established method for BA and DHA in rice noodles has been proved to be suitable for the qualitatively screening of BA and IBA in GVC enrichment broth, potato glucose agar and rice, which could facilitate a rapid and accurate judgement of whether the isolated strain produces BA. Finally, it was observed that BA converted to IBA in samples and the ratio of IBA to BA in the sample is proportional to the duration of contamination (poisoning), which can be used as indirect evidence for tracing the source of the BA. This work contributes to the safety assessment of the entire production process of rice-derived products, addressing a significant public health concern. The method’s high sensitivity and accuracy enable the detection and screening of these compounds at trace levels, which is crucial for ensuring the safety and quality of rice-derived products.

## Data Availability

The raw data supporting the conclusions of this article will be made available by the authors, without undue reservation.
